# A Longitudinal Study of T2 Mapping Combined With Diffusion Tensor Imaging to Quantitatively Evaluate Tissue Repair of Rat Skeletal Muscle After Frostbite

**DOI:** 10.3389/fphys.2020.597638

**Published:** 2021-01-25

**Authors:** Yue Gao, Zhao Lu, Xiaohong Lyu, Qiang Liu, Shinong Pan

**Affiliations:** ^1^ Department of Radiology, Shengjing Hospital of China Medical University, Shenyang, China; ^2^ Department of Radiology, The First Affiliated Hospital of Jinzhou Medical University, Jinzhou, China

**Keywords:** T2 mapping, diffusion tensor imaging, skeletal muscle, frostbite, histological characterization

## Abstract

**Purpose**: T2 mapping and diffusion tensor imaging (DTI) enable the detection of changes in the skeletal muscle microenvironment. We assessed T2 relaxation times, DTI metrics, performed histological characterization of frostbite-induced skeletal muscle injury and repair, and provided diagnostic imaging biomarkers.

**Design and Methods**: Thirty-six Sprague Dawley rats (200 ± 10 g) were obtained. Thirty rats were used for establishing a skeletal muscle frostbite model, and six were untreated controls. Functional MR sequences were performed on rats on days 0, 3, 5, 10, and 14 (*n* = 6 per time point). Rats were then sacrificed to obtain the quadriceps muscles. Tensor eigenvalues (*λ*1, λ2, and λ3), mean diffusivity (MD), fractional anisotropy (FA), and T2 values were compared between the frostbite model and control rats. ImageJ was used to measure the extracellular area fraction (EAF), muscle fiber cross-sectional area (fCSA), and skeletal muscle tumor necrosis factor *α* (TNF-*α*), and Myod1 expression. The correlation between the histological and imaging parameters of the frostbitten skeletal muscle was evaluated. Kolmogorov–Smirnoff test, Leven’s test, one-way ANOVA, and Spearman coefficient were used for analysis.

**Results:** T2 relaxation time of frostbitten skeletal muscle was higher at all time points (*p* < 0.01). T2 relaxation time correlated with EAF, and TNF-*α* and Myod1 expression (*r* = 0.42, *p* < 0.05; *r* = 0.86, *p* < 0.01; *r* = 0.84, *p* < 0.01). The average tensor metrics (MD, *λ*1, λ2, and λ3) of skeletal muscle at 3 and 5 days of frostbite increased (*p* < 0.05), and fCSA correlated with *λ*1, λ2, and λ3, and MD (*r* = 0.65, *p* < 0.01; *r* = 0.48, *p* < 0.01; *r* = 0.52, *p* < 0.01; *r* = 0.62, *p* < 0.01).

**Conclusion:** T2 mapping and DTI imaging detect frostbite-induced skeletal muscle injury early. This combined approach can quantitatively assess skeletal muscle repair and regeneration within 2 weeks of frostbite. Imaging biomarkers for the diagnosis of frostbite were suggested.

## Introduction

The classification of cold-exposure injuries is based on the depth of tissue involved in the injury, which is divided into four levels. Skeletal muscle frostbite belongs to grade 4 frostbite, which is the most serious form of frostbite (Ingram and Raymond, 2013). Frostbite-induced pathological changes, such as cellular edema, microcirculation disorders, and inflammation in skeletal muscle tissue can cause severe sensory dysfunction, amputation, or death. Although muscle frostbite can lead to lifelong disability and even death, it does not attract as much academic interest as other muscle injuries. Clinicians lack precise diagnostic criteria for the extent and degree of frostbite in patients (Petrone et al., 2014). Surgeons may need weeks or months to wait for a clear boundary between living tissue and necrotic tissue to form before performing amputation (Woo et al., 2013).

Assessing the extent of frostbite through imaging increases the possibility of early surgical removal of necrotic tissue (Murphy et al., 2000). At present, multi-phase bone scans constitute the main diagnostic imaging method for the evaluation of frostbitten soft tissue and skeletal muscle viability (Millet et al., 2016). As multi-phase bone scanning requires injection of pertechnetate, contrast media metabolism and resulting side effects may increase the patient’s burden. Another limitation of multiphase bone scans is the poor anatomical resolution of images (Manganaro et al., 2019). MRI is a non-invasive technique, which does not employ ionizing radiation. Functional MRI not only reveals anatomical abnormalities, but also reflects the physiological state of the soft tissue. T2 mapping can be used for the quantitative evaluation of the degree of muscle activation and inflammatory edema under normal physiological and pathological conditions (Meyer and Prior, 2000; Kuo and Carrino, 2007). The transverse relaxation time is represented by the T2 value, which is reflected by the change in signal intensity of the MRI. Acute activity or inflammation will cause the T2 value to rise. In clinical trials, T2 mapping can quantitatively evaluate skeletal muscle injury and myocardial infarction (Zhang et al., 2011; Radunski et al., 2017; Fu et al., 2019). Diffusion tensor imaging (DTI) parameters include three eigenvalues (*λ*1, λ2, and λ3), fractional anisotropy (FA), and mean diffusivity (MD). The three eigenvalues indicate the direction of water diffusion, while FA describes the anisotropy of diffusion. DTI parameters have been used to quantitatively evaluate skeletal muscle injury in runners as well as microenvironmental changes in the skeletal muscle of athletes (Froeling et al., 2015; Keller et al., 2020). In addition, DTI can be used to evaluate the changes in muscle and extracellular matrix microstructure through modeling (Sinha et al., 2020). Overall, functional MRI has good potential for the evaluation of skeletal injury.

The current study aimed to explore the value of T2 mapping and DTI parameters for the noninvasive evaluation of skeletal muscle in a rat model of frostbite and to provide imaging biomarkers for the clinical diagnosis and treatment of patients with severe frostbite.

## Materials and Methods

Experiments were performed under a project license (NO.2019PS468K) granted by Ethics Committee of the institute and was conducted according to the recommendations of the “Guidelines for the Care and Use of Laboratory Animals.”

### Animal Model

**Figure 1:**
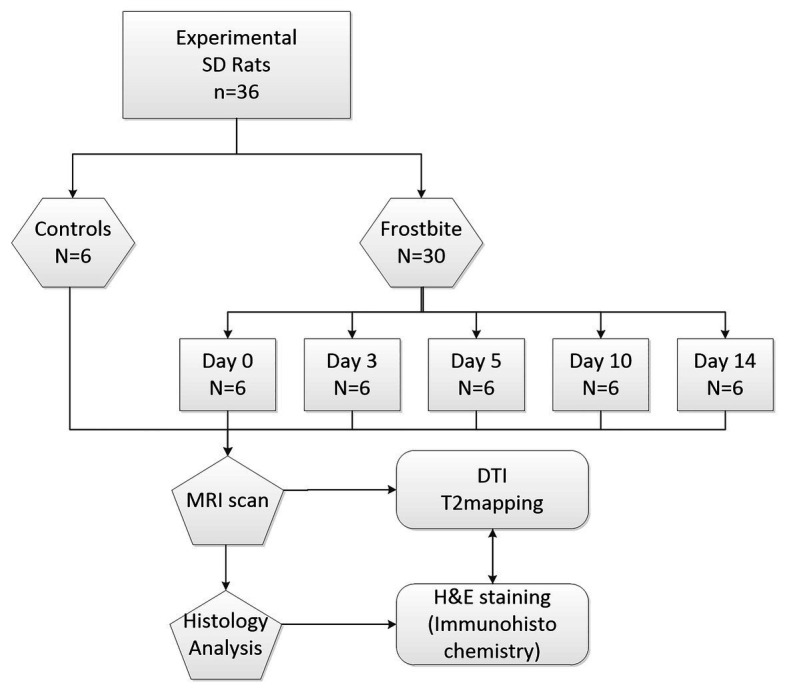
Flowchart demonstrating the study design.

The experimental animals were 36 Sprague Dawley (SD) rats weighing 200 ± 10 g (age: 6 weeks). After the SD rats were numbered, they were randomly divided into two groups, namely the control group (*n* = 6) and the experimental group (*n* = 30). The 30 rats in the experimental group were then randomly divided into five subgroups (six rats/subgroup). Rats in each group underwent frostbite induction followed by functional MRI sequence scans at different time points (0, 3, 5, 10, and 14 days). Immediately afterwards, the quadriceps femoris was taken and fixed with 4% paraformaldehyde solution. The experimental procedure is indicated in Figure 1.

Before the experiment, rats received analgesia and anesthesia. Intraperitoneal injection of the analgesic ibuprofen solution (60 mg/kg) was followed by an intraperitoneal injection of pentobarbital sodium (50 mg/kg). After anesthesia, both lower limbs of rats were shaved, exposing the skin of both lower limbs. Rats were then fixed. A 3 cm long and 1 cm thick dry ice stick (−78.5°C) was taken with an iron clip to tightly touch the bare skin of the rat’s lower limb for 2.5 min to induce frostbite. After the treatment, rats were put back into the cage, and their physiological state was observed. After induction of frostbite, rats were injected with 60 mg/kg of ibuprofen twice a day until the third day after injury. The six rats of the control group did not undergo frostbite treatment.

### MRI Scan

MRI scans were acquired on a 3 Tesla scanner (Ingenia, Philips, software). The elbow joint coil was used to obtain the image. Prior to the MRI scan, rats received injection of the analgesic ibuprofen solution (60 mg/kg) and pentobarbital sodium (50 mg/kg). Rats were placed in the coil in the prone position so that the femur was located in the center of the coil. The imaging sequence included conventional axial T1, sagittal, and coronal images, and the scan range included the entire femur. T2 mapping and DTI sequences were acquired using the same field-of-view (FOV) and geometry. Scan sequence parameters are shown in Table 1. After the MRI scan, rats were sacrificed by intraperitoneal injection of pentobarbital sodium (200 mg/kg).

**Table 1:** Sequence parameters for T1, diffusion tensor imaging (DTI), and T2 mapping.

Sequence	Plane	FOV	Voxel size	Flip angle (°)	TR (ms)	TE (ΔTE; ms)
T1	Axial	100 × 120 × 60	0.33 × 0.37 × 3	90	500	10
DTI	Axial	120 × 90 × 60	1.88 × 2.25 × 2.50	90	2,500	62
T2 mapping	Axial	90 × 121 × 39	0.55 × 0.76 × 3	90	1,500	9–81(9)

DTI, diffusion tensor imaging; T1, Longitudinal relaxation time; T2, transverse relaxation times; FOV, Field of view; TE, echo time; TR, repetition time.

### Image Data Analysis

**Figure 2:**
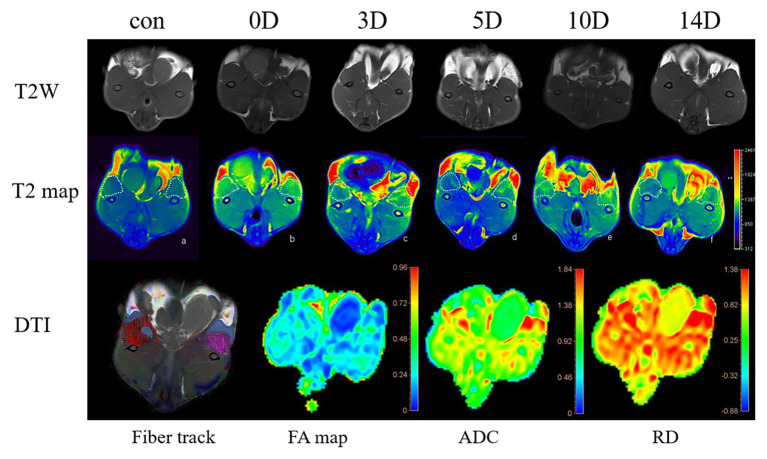
T2WI, T2map axial sequence images in the (a-f) control group and frostbite group (0, 3, 5, 10, 14 days). The muscles in the ROI were manually drawn on the T2 map: the muscle in the control ROI is blue. The color of the ROI area changed significantly after frostbite. The diffusion tensor imaging (DTI) sequence: the schematic diagram of the fiber track of the DTI and the corresponding fractional anisotropy (FA) map, mean diffusivity (MD) map, and RD map. The area with fewer muscle fibers in the fiber track diagram is consistent with the high-signal area in the MD picture.

Two observers with experience in MR image analysis (Y. Gao and XH. Lyu, with 6 and 10 years of MR diagnosis experience, respectively) assessed the MR images with an assessment interval of 4 weeks. They were blind to image information when analyzing the images. To evaluate the validity of MRI measurements, test-retest reliability was analyzed. Interobserver and intra-observer reliabilities for the imaging parameters were analyzed using the intraclass correlation coefficient (ICC). The original images were imported into the Philips post-processing workstation and analyzed by the workstation function tool software. T2 mapping pseudo color images were automatically generated after scanning. Fiber track was implemented by workstation post-processing. The areas of interest were recognized by the observers and drawn manually (Figure 2).

### Histological Examination

#### Hematoxylin-Eosin Staining

After the MRI scan, animals were euthanized using 100% carbon dioxide. Skin was cut off the rat’s lower limbs, and the quadriceps muscle was separated, removed, soaked in 4% paraformaldehyde solution, and then fixed at room temperature for 1 week. After fixing, the sample was dehydrated, permeated with gradient alcohol and xylene, and embedded in paraffin. The paraffin block was cut into horizontal tissue sections (3 μm), and slices were heated at 70°C for 4 h. Paraffin sections were hematoxylin-eosin (HE) stained with an automatic cylinder passing machine.

#### Immunohistochemistry

The tissue sections were dewaxed, and Tris-EDTA repair solution was used to repair them. Sections were then immersed in endogenous peroxidase blocker for 30 min, followed by a wash with PBS. Sections were incubated with serum for 30 min at room temperature, followed by incubation with primary antibodies against TNF-α (1:200) and Myod1 (1:200) overnight at 4°C, or overnight incubation with PBS as a negative control. Sections were then rewarmed at room temperature for 1 h, washed with PBS, and incubated with the appropriate secondary antibody at room temperature for 25 min. After the secondary antibody was washed away, sections were incubated with peroxidase at room temperature for 25 min, and the DAB kit was used to carry out the color reaction.

Slices were sealed with gum, followed by observation and image collection under a microscope.

The Image J software was used to analyze the extracellular matrix area fraction (EAF) and fiber cross-sectional area (fCSA) of HE images as well as the expression levels of targeted proteins in histochemical images. We use Image J software to open the HE image of skeletal muscle tissue and adjust the image mode to RGB stack mode. Use the “threshold” function in the “Adjust” module to automatically identify muscle fibers, and use the “Measure” function to measure the percentage of muscle fiber area (fCSA%). EAF = 100% − fCSA%. fCSA is to measure the cross-sectional area of a single muscle fiber by manually contouring and measuring after the image J software identifies the muscle fiber. Six images were collected for each subgroup, and 10 muscle fCSA were collected for each image, and the average value was taken to obtain the fCSA of each subgroup.

#### Western Blot

Western blotting was performed using standard protocols. Extract total protein from skeletal muscle tissue and mix it with 5× loading buffer at a ratio of 4:1. Equal amounts of protein were separated by 10% sodium dodecyl sulfate-polyacrylamide gel electrophoresis (SDS-PAGE) and transferred to polyacrylamide difluoride (PVDF) membranes. After blocking with 5% skimmed milk for 2 h at room temperature, the membrane was combined with anti-Myod1 (dilution 1:1,000, catalog number 18943-1-AP, Proteintech), anti-TNF-*α* (dilution 1: 1,000, product catalog number 17950-1-AP, Proteintech), and anti-GAPDH (dilution 1:5,000, catalog number 60004-1-Ig, Proteintech), and then gently shake at 4°C overnight. On the second day, the Myod1 membrane and TNF-α membrane were incubated with horseradish peroxidase-conjugated goat anti-rabbit IgG antibody (dilution 1:5,000; catalog number SA00001-2, Proteintech) for 2 h at room temperature. GAPDH membrane and horseradish peroxidase-conjugated goat anti-mouse IgG antibody (dilution 1:5,000; catalog number SA00001-1, Proteintech) were incubated for 2 h at room temperature, and then washed PVDF membranes in TBST buffer (10 mM Tris/HCl, 150 mM NaCl, and 0.05% Tween-20, pH 7.5) three times, and developed using enhanced chemiluminescence reagents (NCM Biotech). The Image J software was used to analyze densitometry values and standardized to GAPDH.

### Statistical Analysis

The normality of distributions was tested using the Kolmogorov–Smirnov test and normal Q-Q plots. For quantitative variables that were normally distributed, the data are expressed as mean ± SD. Leven’s test was used to check the homogeneity of variance. One-way ANOVA was used to compare differences in DTI and T2 mapping parameters between groups, and the Bonferroni correction was employed to adjust the *p*-value for multiple comparisons. The Spearman correlation coefficient was used to analyze the correlation between TNF-α and Myod1 expression, EAF, and T2 values. The Spearman correlation coefficient was also used to analyze the correlation between fCSA, *λ*1, λ2, λ3, MD, and FA. Histological parameters (TNF-α, Myod1, EAF, and fCSA) were treated as independent variables, while imaging parameters (T2 value, λ1, λ2, λ3, MD, and FA) were treated as dependent variables. Statistical significance was established at *p* < 0.05. Statistical analyses were performed using SPSS (Version 22.0; SPSS Inc., Chicago, IL).

## Results

### Morphological Changes of Skeletal Muscle Tissue Within 2 Weeks of Frostbite

**Figure 3:**
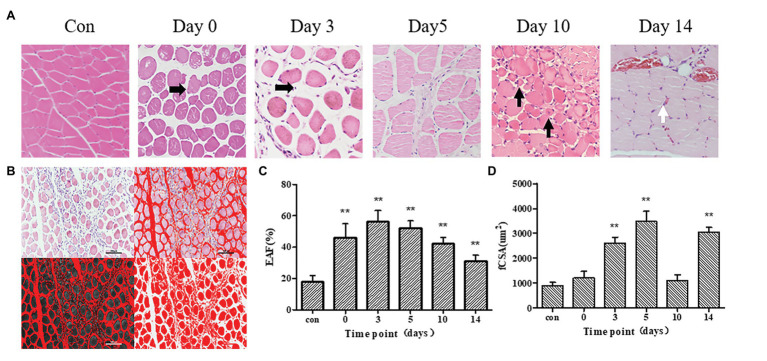
**(A)** Hematoxylin-eosin (HE) stained images in control group, 0, 3, 5, 10, and 14 days after frostbite. Damaged skeletal muscle tissue exhibits diffuse swelling of muscle cells or inflammatory cells infiltration (Horizontal black arrow). On day 10, regenerated skeletal muscle (Vertical black arrow) was observed. On day 14, red blood cells (white arrows) can be observed in muscle tissue. **(B)** This example shows how to measure the fiber cross-sectional area (fCSA) and extracellular area fraction (EAF). **(C,D)**: EAF and fCSA of control group and frostbite groups. Scale bar, 200 μm. EAF, extracellular matrix area fraction; fCSA, fiber cross-sectional area. ^**^ *p* < 0.01.

At day 0 after frostbite, skeletal muscle cells exhibited edema, the intercellular space expanded, and interstitial components increased. Subsequently, inflammatory cell infiltration increased, clearing necrotic muscle cells. Inflammatory infiltration persisted until about the tenth day. Ten days after frostbite, there were more new muscle fibers in the remodeled skeletal muscle tissue, and these were irregular in shape. At day 14 after frostbite, skeletal muscle tissue still exhibited blood cell deposition and expression of inflammatory factors (Figure 3A).

The schematic diagram of measuring fCSA and EAF is shown in (Figure 3B). The skeletal muscle cells of control group rats were closely arranged with less interstitial components. The fCSA values of the frostbite group skeletal muscle at days 3, 5, and 14 were significantly different from those of the control group skeletal muscle (all *p* < 0.01; Table 2; Figures 3C,D). Further, fCSA was correlated with *λ*1, λ2, λ3, and MD (*r* = 0.65, *p* < 0.01, *r* = 0.48, *p* < 0.01, *r* = 0.52, *p* < 0.01, and *r* = 0.62, *p* < 0.01, respectively). EAF decreased 10 days after frostbite and was significantly different from the EAF at 5 days after frostbite (*p* = 0.01). At 14 days after frostbite, EAF was significantly different from that of the control group (*p* = 0.01). The EAF of skeletal muscle within 5 days of frostbite was strongly positively correlated with the T2 value (*r* = 0.80, *p* < 0.01), while EAF within 2 weeks of frostbite was only moderately correlated with the T2 value (*r* = 0.42, *p* < 0.05; Figure 3C).

**Table 2:** EAF and fCSA in frostbite and control.

Parameter	control (*N* = 6)	frostbite				
		0D (*N* = 6)	3D (*N* = 6)	5D (*N* = 6)	10D (*N* = 6)	14D (*N* = 6)
EAF(%)	18(3.85)	46(8.93)^**^	56(7.31)^**^	52(4.79)^**^	42(4.26)^**^	31(3.88)^**^
fCSA(um2)	895(131)	1,202(266)	2,593(248)^**^	3,473(409)^**^	1,103(218)	3,035(197)^**^

Data are given as mean (SD).

** *p* < 0.01.

EAF, extracellular matrix area fraction; fCSA, fiber cross-sectional area.

### Changes in the Relative Expression Levels of TNF-*α* and Myod1 in Frostbitten Skeletal Muscle

**Figure 4:**
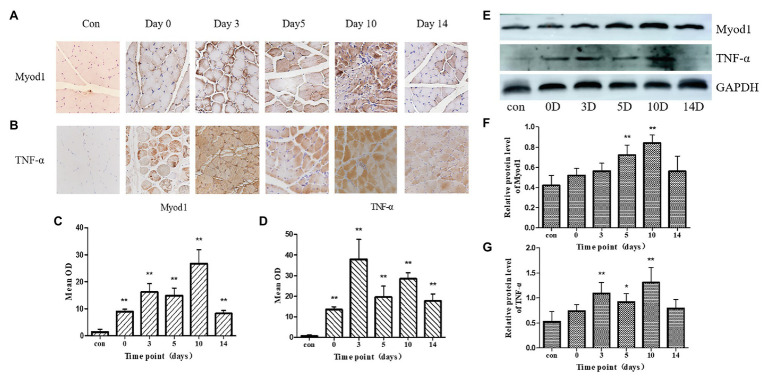
**(A,B)**: immunohistochemical pictures of Myod1 and TNF-*α* at control and frostbite (0, 3, 5, 10, and 14 days). **(C,D)**: mean optical density (OD) of Myod1 and TNF-α in control group and frostbite groups. **(E)** Western blot pictures of Myod1 and TNF-α. **(F,G)**: protein expression of Myod1 and TNF-α in control group and frostbite groups. Scale bar, 200 μm. OD, optical density. ^*^p < 0.05, ^**^p < 0.01.

The TNF-α and Myod1 values of each group followed a normal distribution. For the expression levels of TNF-α and Myod1, the results of immunohistochemistry and western blot are in good agreement (Myod1, *r* = 0.72, *p* < 0.01, TNF-α, *r* = 0.66, *p* < 0.01). Within 2 weeks of frostbite, there were two peaks in skeletal muscle TNF-α expression. These peak values were observed at day 3 and day 10 (Figure 4). On the 14th day after frostbite, the relative expression of TNF-α was still significantly higher than that in the control group (*p* < 0.01). The relative expression of TNF-α was positively correlated with the T2 value (*r* = 0.86, *p* < 0.01). Myod1 expression in day 10 of frostbite was significantly higher than in the control group (*p* < 0.01). Further, Myod1 expression remained higher than in the control group at 14 days of frostbite (*p* < 0.01). The relative expression of Myod1 was positively correlated with the T2 value (*r* = 0.84, *p* < 0.01).

### Dynamic Changes in MR Imaging of Frostbitten Skeletal Muscle

**Figure 5:**
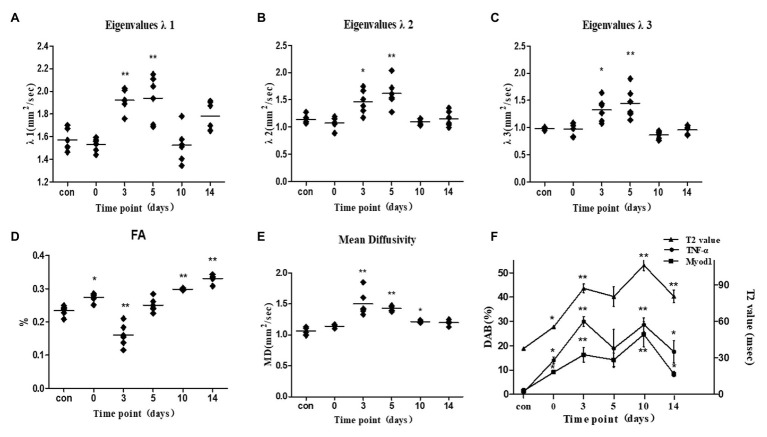
**(A–F)** Frostbite skeletal muscle eigenvalues *λ*1, λ2, λ3, FA, MD, and T2 at different time points. Compared with the control group, the frostbite muscle MD and λ1, λ2, and λ3 increased from 3 to 5 days, and FA decreased. The last picture is a schematic diagram of the T2 value, TNF-α, and Myod1. The three curves represent relative expression levels of T2 values, TNF-α, and Myod1. The T2 values show a bimodal change. ^*^p < 0.05, ^**^p < 0.01.

Intraclass correlation coefficient findings revealed that the reliability of MR imaging was substantial or excellent (from 0.87 to 0.96), except for FA (0.61–0.76; Table 3). The T2 values and DTI parameters of the control group and the experimental group are shown in Table 4. The T2 value of each group conformed to normal distribution. The T2 value of frostbitten skeletal muscle was higher than that of the control group at all time points. The first peak of the T2 value was observed on the third day after frostbite. Thereafter, the T2 value decreased, and there was no significant difference between the T2 value on the fifth day and that on the third day (*p* > 0.05). The second peak was observed on the tenth day after frostbite. The skeletal muscle T2 value on day 14 after frostbite remained higher than that of the control group (*p* < 0.01). Further, the T2 value of the frostbitten skeletal muscle exhibited a “bimodal” change (Figure 5).

**Table 3:** Interobserver reliability and intra-observer reliability of T2 value and DTI parameters.

	Interobserver reliability	Intra-observer reliability	
		Observer 1	Observer 2
T2 value	0.95	0.92	0.90
λ1–3	0.96	0.95	0.90
FA	0.76	0.71	0.61
MD	0.96	0.90	0.87

Interobserver and intra-observer reliabilities for T2 value and DTI parameters calculated using intraclass correlation coefficient. λ1–3, three tensor eigenvalues; FA, fractional anisotropy; MD, mean diffusivity.

**Table 4:** Mean diffusion tensor parameters and T2 values in frostbite and control.

Parameter	control (*N* = 6)	frostbite				
		0D (*N* = 6)	3D (*N* = 6)	5D (*N* = 6)	10D (*N* = 6)	14D (*N* = 6)
λ1(mm^2^/s)	1.57(0.10)	1.53(0.06)	1.92(0.10)^*^	1.94(0.20)^*^	1.52(0.17)	1.78(0.13)^*^
λ2(mm^2^/s)	1.14(0.07)	1.08(0.11)	1.46(0.22)^*^	1.62(0.25)^*^	1.10(0.06)	1.15(0.14)
λ3(mm^2^/s)	0.98(0.02)	0.97(0.12)	1.33(0.21)^*^	1.45(0.28)^*^	0.87(0.08)	0.96(0.07)
MD(mm^2^/s)	1.06(0.06)	1.14(0.03)	1.50(0.19)^*^	1.43(0.05)^*^	1.22(0.02)^*^	1.20(0.05)
FA	0.23(0.01)	0.27(0.02)^*^	0.25(0.02)^*^	0.25(0.02)	0.30(0.00)^*^	0.33(0.02)^*^
T2 (ms)	37.6(0.9)	55.5(1.4)^*^	87.1(3.6)^*^	80.4(8.1)^*^	106.1(4.5)^*^	80.4(5.2)^*^

Data are given as mean (SD).

* *p* < 0.05.

λ1–3, three tensor eigenvalues.

The MD, FA, and three eigenvalues of the control group and frostbite model were measured on the DTI sequence (Table 4). Three days after frostbite, MD and eigenvalues were higher (*p* < 0.01, *p* < 0.01, *p* < 0.05, *p* < 0.05), while FA was lower (*p* < 0.01). The peak of MD and the three eigenvalues was observed at 3–5 days after frostbite. Five days after frostbite, MD, FA, and eigenvalues returned to normal levels (*p* ˃ 0.05). On day 14 after frostbite, FA appeared elevated (*p* < 0.01).

## Discussion

In the current study, we quantitatively described the dynamic changes occurring in the skeletal muscle microenvironment over a critical 2-week period after frostbite. The current study confirmed the use of DTI combined with T2 mapping for the quantitative evaluation of muscle tissue repair after frostbite. The main findings were as follows: (1) The T2 value reflected muscle damage and its extent after frostbite and showed a bimodal change; (2) The eigenvalues *λ*1–λ3 and MD of the frostbitten skeletal muscle tissue were higher, reaching peak values around 3–5 days after frostbite, accompanied by an increase in λ1, λ2, λ3, and MD, as well as a decrease in FA; and (3) The T2 value and DTI parameters were correlated with pathological changes in the frostbitten skeletal muscle.

### T2 Value as an Indicator of Skeletal Muscle Damage and Repair

Skeletal muscle frostbite is characterized by the direct or indirect cell damage mediated by low temperature conditions, causing secondary vascular microcirculation disorders and inflammation in skeletal muscle tissue (Dana et al., 1969; Quinn, 1985; Imray et al., 2009; Macmillan and Sinclair, 2011). The dynamic balance between inflammation and muscle regeneration affects the prognosis of skeletal muscle frostbite. Skeletal muscle injury mainly goes through three stages, namely damage, repair, and remodeling (Hurme et al., 1991). Repair following skeletal muscle injury follows a relatively constant pattern (Filippin et al., 2009). In our study, it took approximately 2 weeks for skeletal muscle to enter the remodeling stage after frostbite. The T2 value of skeletal muscle increased immediately after frostbite and showed a bimodal change. This change was similar to the observations of Fernández and colleagues in myocardial infarction (Fernandez-Jimenez et al., 2015). They reported that the first peak was caused by myocardial ischemia-reperfusion, and the second peak represented the process of myocardial tissue repair. In our study, the two peaks in the T2 value of the skeletal muscle following frostbite appeared later than those of myocardial infarction. The peak T2 value of the skeletal muscle following frostbite was higher than the T2 values following skeletal muscle strain and contusion (Zhang et al., 2011; Fu et al., 2019). While frostbite is similar to other skeletal muscle injury, the degree of injury and the inflammatory response are more severe. Based on the current observations, the T2 value can sensitively detect muscle damage after frostbite.

During skeletal muscle frostbite, the interstitial composition of skeletal muscle tissue changes greatly. In this study, the T2 value strongly correlated with the EAF of the skeletal muscle tissue up to 5 days after frostbite. The increase in the free water in the interstitial space of the skeletal muscle cells prolongs the T2 relaxation time, resulting in an increased T2 value (Patten et al., 2003). The T2 value during the early frostbite period can accurately reflect the interstitial inflammatory edema, and inflammation of muscle tissue is the main pathological process at this stage. Five days after frostbite, the correlation between the EAF and the T2 value was poor. This phenomenon indicates that during the later period of repair following skeletal muscle frostbite, interstitial edema is not the main pathological change, and changes in muscle cells are predominant. A reason for the decrease in EAF and the increase in the T2 value 5 days after frostbite may be: (1) the accumulation of metabolites in muscle cells causing an increase in the osmotic pressure in cells and changing the mobility of water in myofibrils (Fleckenstein et al., 1991); (2) the T2 value is affected not only by the interstitial, but also by the intracellular composition, for example, through changes in protein concentration.

### DTI Parameters as Indicators of the State of the Frostbitten Skeletal Muscle

Compared to T2-weighted imaging, DTI parameters can more sensitively detect changes in the muscle microenvironment (Giraudo et al., 2018). The increase in MD and decrease in FA after muscle injury represent a reduction in the limitation of water diffusion (Zaraiskaya et al., 2006; Yanagisawa et al., 2011). Further, the increase in MD and decrease in FA are associated with pathological changes, such as damaged cell swelling, interstitial edema, or destruction of the diffusion barrier (Froeling et al., 2015). In our study, MD and the three eigenvalues increased after frostbite, while FA decreased. The current results are similar to those reported by Zaraiskaya et al. (2006). This phenomenon mainly occurs within 3–5 days of frostbite, indicating that the barrier of frostbitten skeletal muscle tissue is extensively damaged, the cells are swollen, and interstitial edema is severe. In our study, *λ*1, λ2, λ3, and MD were sensitive to changes in the skeletal muscle microenvironment and were closely related to the fCSA. This is similar to observations by Berry et al. (2018). λ1 corresponds to the water diffusion state parallel to the long axis of the muscle fibers (Damon et al., 2002). The increase of eigenvalues λ1–3 indicates that water molecules in the muscle are easily spread in all directions as a result of the extensive swelling and rupture of skeletal muscle fibers following frostbite. Ten days after frostbite, λ2, λ3, and MD decreased to normal levels, while λ1 remained elevated, indicating that the sarcolemma of the newly formed muscle fibers was intact, but their long axes were still broken. At this point, skeletal muscle is at the stage of remodeling and regeneration. DTI parameters reflect water diffusion within frostbitten skeletal muscle but are not sensitive indicators of inflammation and skeletal muscle regeneration. This is consistent with the findings of Froeling lab (Froeling et al., 2015). The combination of T2 value and DTI parameters can comprehensively determine the survival status of frostbitten skeletal muscle and provide good imaging evidence for assessing the degree and extent of frostbite.

### The Relationship Between the T2 Value, Inflammation, and Regeneration

In this study, the peak of the inflammatory factor TNF-α expression in the frostbitten skeletal muscle tissue was basically in parallel with the peak of the T2 value (Figure 4). Research has shown that on the third day after skeletal muscle injury, satellite cells are activated, and muscle tissue begins to regenerate (Jarvinen et al., 2005). The strong inflammatory response and initial muscle regeneration produced the first peak T2 value following frostbite. Myod1 plays an important role in the myogenic differentiation of skeletal muscle (Sabourin et al., 1999). On day 10 after frostbite, a large number of regenerate muscle fibers appeared, which was the result of the activity of Myod1 and other myogenic factors. There are many central nuclei in the regenerated muscle fiber, which is consistent with the characteristics of the regenerated muscle in other studies (Zhao et al., 2019; Mosele et al., 2020). Myod1 can regulate the transformation of muscle fiber types, which is accompanied by metabolic changes (Hughes et al., 1997; Maves et al., 2007). Thus, the increased expression of Myod1 is associated with the fiber type transformation of the regenerating muscle tissue (Talbot and Maves, 2016). The peak of Myod1 indicates that the regenerated muscle enters the remodeling stage. At the same time on day 10 of frostbite, the expression level of TNF-α in skeletal muscle reached a second peak. Muscle regeneration occurs in an environment with high levels of inflammation. Further, inflammation is considered to be a critical response required for muscle regeneration after muscle injury (Tidball, 1995, 2005), and TNF-α is a key cytokine involved in the inflammatory response during skeletal muscle regeneration. Studies have suggested that, to a certain extent, the inflammatory response promotes skeletal muscle regeneration (Cantini et al., 2002; Tidball and Wehling-Henricks, 2007). Interestingly, TNF-α is a key mediator of myogenic differentiation and plays an important role in the regulation of cell cycle exit and the initiation of myogenic differentiation in satellite cells (Li et al., 2014). Thus, as expected, the second peak of Myod1 and TNF-α expression promoted the differentiation and remodeling of the skeletal muscle after frostbite. This is consistent with previous research results (Warren et al., 2002). The regeneration, remodeling, and inflammation of frostbitten skeletal muscle also resulted in a second peak in the T2 value. The decrease in the T2 value after 10 days of frostbite indicated that skeletal muscle had entered the remodeling stage. The balance between inflammation and regeneration determines the time required for the T2 value to return to normal levels, as well as the time for skeletal muscle repair (Tidball, 2005). Fourteen days after frostbite, the T2 value and TNF-α expression in rat skeletal muscle were still higher than normal, meaning that inflammation was still ongoing in the skeletal muscle. Of note, excessive inflammation depletes muscle satellite cells and hinders muscle regeneration. T2 values in frostbitten muscle remained high, indicating poor prognosis.

## Limitations

At present, pathological indicators reflect the state of the inflammatory response in frostbitten skeletal muscle. However, the detection of pathological indicators is relatively simple, and the activity of inflammatory cells during skeletal muscle repair needs to be further explored in order to provide an effective target for the treatment of frostbite. In addition, there is little discussion about the correlation between DTI parameters and pathological status, and the relationship between the cross-sectional area of muscle fibers and the DTI parameters should be further studied. Studies have found that different tracking parameter ranges have different effects on muscle diffusion parameters (Forsting et al., 2020). To reduce bias, all experimental images were measured using the same fiber tracking stop criterion. DTI parameters are known to be affected by muscle contraction and stretching, resulting in changes in *λ*2, λ3, MD, and FA (Schwenzer et al., 2009), compromising the diagnostic performance of DTI with regard to the degree of muscle damage. In this study, we scanned the DTI sequence after anesthetizing rats and performed uniform positioning. Muscle contraction and extension have little effect on the results of DTI parameter measurement in our study. In our study, no conventional imaging methods were used to assess skeletal muscle frostbite. The comparative study of conventional imaging and functional imaging can more comprehensively reflect the pathological state of skeletal muscle frostbite and improve the accuracy of diagnosis, which should be further studied.

## Conclusion

This study assessed the imaging and pathological features of frostbite-induced skeletal muscle injury, repair, and regeneration, and verified the correlation between imaging parameters and pathological indicators. The T2 value can reflect skeletal muscle frostbite from the early stage, and distinguish between the different stages of frostbite repair (inflammation and regeneration), indicating the clinical outcome of frostbite skeletal muscle. DTI reflected the muscle fiber diameter and the state of water diffusion in frostbitten skeletal muscle, but was not sensitive to skeletal muscle inflammation. The T2 value and DTI parameters can be used together as imaging biomarkers to assess the prognosis of frostbite and provide a basis for the clinical treatment of severe frostbite at different stages. Functional MRI longitudinal quantitative evaluation of skeletal muscle frostbite provides new insights for the clinical treatment of severe frostbite.

## Data Availability Statement

The original contributions presented in the study are included in the article/Supplementary Material, further inquiries can be directed to the corresponding author.

## Ethics Statement

The animal study was reviewed and approved by Ethics Committee of Shengjing Hospital of China Medical University.

## Author Contributions

YG and SP conceived and designed the study. YG and XL carried out the experiments. YG and ZL analyzed the data and prepared the figures. YG, ZL, and QL interpreted the results of the experiments. YG drafted the manuscript. ZL and SP edited and revised the manuscript. All authors contributed to the article and approved the submitted version.

### Conflict of Interest

The authors declare that the research was conducted in the absence of any commercial or financial relationships that could be construed as a potential conflict of interest.

## References

[ref1] Berry D. B. Regner B. Galinsky V. Ward S. R. Frank L. R. (2018). Relationships between tissue microstructure and the diffusion tensor in simulated skeletal muscle. Magn. Reson. Med. 80, 317–329. 10.1002/mrm.26993, PMID: 29090480 PMC5876103

[ref2] Cantini M. Giurisato E. Radu C. Tiozzo S. Pampinella F. Senigaglia D.. (2002). Macrophage-secreted myogenic factors: a promising tool for greatly enhancing the proliferative capacity of myoblasts in vitro and in vivo. Neurol. Sci. 23, 189–194. 10.1007/s100720200060, PMID: 12536288

[ref3] Damon B. M. Ding Z. Anderson A. W. Freyer A. S. Gore J. C. (2002). Validation of diffusion tensor MRI-based muscle fiber tracking. Magn. Reson. Med. 48, 97–104. 10.1002/mrm.10198, PMID: 12111936

[ref4] Dana A. S. Jr. Rex I. H. Jr. Samitz M. H. (1969). The hunting reaction. Arch. Dermatol. 99, 441–450. 5769328

[ref5] Fernandez-Jimenez R. Sanchez-Gonzalez J. Aguero J. Garcia-Prieto J. Lopez-Martin G. J. Garcia-Ruiz J. M.. (2015). Myocardial edema after ischemia/reperfusion is not stable and follows a bimodal pattern: imaging and histological tissue characterization. J. Am. Coll. Cardiol. 65, 315–323. 10.1016/j.jacc.2014.11.004, PMID: 25460833

[ref6] Filippin L. I. Moreira A. J. Marroni N. P. Xavier R. M. (2009). Nitric oxide and repair of skeletal muscle injury. Nitric Oxide 21, 157–163. 10.1016/j.niox.2009.08.002, PMID: 19682596

[ref7] Fleckenstein J. L. Haller R. G. Lewis S. F. Archer B. T. Barker B. R. Payne J.. (1991). Absence of exercise-induced MRI enhancement of skeletal muscle in McArdle's disease. J. Appl. Physiol. 71, 961–969. 10.1152/jappl.1991.71.3.961, PMID: 1757335

[ref8] Forsting J. Rehmann R. Froeling M. Vorgerd M. Tegenthoff M. Schlaffke L. (2020). Diffusion tensor imaging of the human thigh: consideration of DTI-based fiber tracking stop criteria. MAGMA 33, 343–355. 10.1007/s10334-019-00791-x, PMID: 31776703

[ref9] Froeling M. Oudeman J. Strijkers G. J. Maas M. Drost M. R. Nicolay K.. (2015). Muscle changes detected with diffusion-tensor imaging after long-distance running. Radiology 274, 548–562. 10.1148/radiol.14140702, PMID: 25279435

[ref10] Fu C. Xia Y. Meng F. Li F. Liu Q. Zhao H.. (2019). MRI quantitative analysis of eccentric exercise-induced skeletal muscle injury in rats. Acad. Radiol. 27, e72–e79. 10.1016/j.acra.2019.05.011, PMID: 31300358

[ref11] Giraudo C. Motyka S. Weber M. Karner M. Resinger C. Feiweier T.. (2018). Normalized STEAM-based diffusion tensor imaging provides a robust assessment of muscle tears in football players: preliminary results of a new approach to evaluate muscle injuries. Eur. Radiol. 28, 2882–2889. 10.1007/s00330-017-5218-9, PMID: 29423575 PMC5986840

[ref12] Hughes S. M. Koishi K. Rudnicki M. Maggs A. M. (1997). MyoD protein is differentially accumulated in fast and slow skeletal muscle fibres and required for normal fibre type balance in rodents. Mech. Dev. 61, 151–163. 10.1016/s0925-4773(96)00631-4, PMID: 9076685

[ref13] Hurme T. Kalimo H. Lehto M. Jarvinen M. (1991). Healing of skeletal muscle injury: an ultrastructural and immunohistochemical study. Med. Sci. Sports Exerc. 23, 801–810. PMID: 1921672

[ref14] Imray C. Grieve A. Dhillon S. The Caudwell Xtreme Everest Research Group (2009). Cold damage to the extremities: frostbite and non-freezing cold injuries. Postgrad. Med. J. 85, 481–488. 10.1136/pgmj.2008.068635, PMID: 19734516

[ref15] Ingram B. J. Raymond T. J. (2013). Recognition and treatment of freezing and nonfreezing cold injuries. Curr. Sports Med. Rep. 12, 125–130. 10.1249/JSR.0b013e3182877454, PMID: 23478565

[ref16] Jarvinen T. A. Jarvinen T. L. Kaariainen M. Kalimo H. Jarvinen M. (2005). Muscle injuries: biology and treatment. Am. J. Sports Med. 33, 745–764. 10.1177/0363546505274714, PMID: 15851777

[ref17] Keller S. Yamamura J. Sedlacik J. Wang Z. J. Gebert P. Starekova J.. (2020). Diffusion tensor imaging combined with T2 mapping to quantify changes in the skeletal muscle associated with training and endurance exercise in competitive triathletes. Eur. Radiol. 30, 2830–2842. 10.1007/s00330-019-06576-z, PMID: 31953666

[ref18] Kuo G. P. Carrino J. A. (2007). Skeletal muscle imaging and inflammatory myopathies. Curr. Opin. Rheumatol. 19, 530–535. 10.1097/BOR.0b013e3282efdc66, PMID: 17917531

[ref19] Li Y. P. Niu A. Wen Y. (2014). Regulation of myogenic activation of p38 MAPK by TACE-mediated TNFalpha release. Front. Cell Dev. Biol. 2:21. 10.3389/fcell.2014.00021, PMID: 25364728 PMC4207040

[ref20] Macmillan H. A. Sinclair B. J. (2011). Mechanisms underlying insect chill-coma. J. Insect Physiol. 57, 12–20. 10.1016/j.jinsphys.2010.10.004, PMID: 20969872

[ref21] Manganaro M. S. Millet J. D. Brown R. K. Viglianti B. L. Wale D. J. Wong K. K. (2019). The utility of bone scintigraphy with SPECT/CT in the evaluation and management of frostbite injuries. Br. J. Radiol. 92:20180545. 10.1259/bjr.20180545, PMID: 30359097 PMC6404829

[ref22] Maves L. Waskiewicz A. J. Paul B. Cao Y. Tyler A. Moens C. B.. (2007). Pbx homeodomain proteins direct Myod activity to promote fast-muscle differentiation. Development 134, 3371–3382. 10.1242/dev.003905, PMID: 17699609

[ref23] Meyer R. A. Prior B. M. (2000). Functional magnetic resonance imaging of muscle. Exerc. Sport Sci. Rev. 28, 89–92. PMID: 10902092

[ref24] Millet J. D. Brown R. K. Levi B. Kraft C. T. Jacobson J. A. Gross M. D.. (2016). Frostbite: spectrum of imaging findings and guidelines for management. Radiographics 36, 2154–2169. 10.1148/rg.2016160045, PMID: 27494386 PMC5131839

[ref25] Mosele F. C. Bissi Ricci R. Abreu P. Rosa Neto J. C. (2020). Muscle regeneration in adiponectin knockout mice showed early activation of anti-inflammatory response with perturbations in myogenesis. J. Cell. Physiol. 235, 6183–6193. 10.1002/jcp.29547, PMID: 32003014

[ref26] Murphy J. V. Banwell P. E. Roberts A. H. McGrouther D. A. (2000). Frostbite: pathogenesis and treatment. J. Trauma 48, 171–178. 10.1097/00005373-200001000-00036, PMID: 10647591

[ref27] Patten C. Meyer R. A. Fleckenstein J. L. (2003). T2 mapping of muscle. Semin. Musculoskelet. Radiol. 7, 297–305. 10.1055/s-2004-815677, PMID: 14735428

[ref28] Petrone P. Asensio J. A. Marini C. P. (2014). Management of accidental hypothermia and cold injury. Curr. Probl. Surg. 51, 417–431. 10.1067/j.cpsurg.2014.07.004, PMID: 25242454

[ref29] Quinn P. J. (1985). A lipid-phase separation model of low-temperature damage to biological membranes. Cryobiology 22, 128–146. 10.1016/0011-2240(85)90167-1, PMID: 3920005

[ref30] Radunski U. K. Lund G. K. Saring D. Bohnen S. Stehning C. Schnackenburg B.. (2017). T1 and T2 mapping cardiovascular magnetic resonance imaging techniques reveal unapparent myocardial injury in patients with myocarditis. Clin. Res. Cardiol. 106, 10–17. 10.1007/s00392-016-1018-5, PMID: 27388331

[ref31] Sabourin L. A. Girgis-Gabardo A. Seale P. Asakura A. Rudnicki M. A. (1999). Reduced differentiation potential of primary MyoD−/− myogenic cells derived from adult skeletal muscle. J. Cell Biol. 144, 631–643. 10.1083/jcb.144.4.631, PMID: 10037786 PMC2132931

[ref32] Schwenzer N. F. Steidle G. Martirosian P. Schraml C. Springer F. Claussen C. D.. (2009). Diffusion tensor imaging of the human calf muscle: distinct changes in fractional anisotropy and mean diffusion due to passive muscle shortening and stretching. NMR Biomed. 22, 1047–1053. 10.1002/nbm.1409, PMID: 19618408

[ref33] Sinha U. Malis V. Chen J. S. Csapo R. Kinugasa R. Narici M. V.. (2020). Role of the extracellular matrix in loss of muscle force with age and unloading using magnetic resonance imaging, biochemical analysis and computational models. Front. Physiol. 11:626. 10.3389/fphys.2020.00626, PMID: 32625114 PMC7315044

[ref34] Talbot J. Maves L. (2016). Skeletal muscle fiber type: using insights from muscle developmental biology to dissect targets for susceptibility and resistance to muscle disease. Wiley Interdiscip. Rev. Dev. Biol. 5, 518–534. 10.1002/wdev.230, PMID: 27199166 PMC5180455

[ref35] Tidball J. G. (1995). Inflammatory cell response to acute muscle injury. Med. Sci. Sports Exerc. 27, 1022–1032. 10.1249/00005768-199507000-00011, PMID: 7564969

[ref36] Tidball J. G. (2005). Inflammatory processes in muscle injury and repair. Am. J. Phys. Regul. Integr. Comp. Phys. 288, R345–R353. 10.1152/ajpregu.00454.2004, PMID: 15637171

[ref37] Tidball J. G. Wehling-Henricks M. (2007). Macrophages promote muscle membrane repair and muscle fibre growth and regeneration during modified muscle loading in mice in vivo. J. Physiol. 578, 327–336. 10.1113/jphysiol.2006.118265, PMID: 17038433 PMC2075127

[ref38] Warren G. L. Hulderman T. Jensen N. McKinstry M. Mishra M. Luster M. I.. (2002). Physiological role of tumor necrosis factor alpha in traumatic muscle injury. FASEB J. 16, 1630–1632. 10.1096/fj.02-0187fje, PMID: 12207010

[ref39] Woo E. K. Lee J. W. Hur G. Y. Koh J. H. Seo D. K. Choi J. K.. (2013). Proposed treatment protocol for frostbite: a retrospective analysis of 17 cases based on a 3-year single-institution experience. Arch. Plast. Surg. 40, 510–516. 10.5999/aps.2013.40.5.510, PMID: 24086802 PMC3785582

[ref40] Yanagisawa O. Kurihara T. Kobayashi N. Fukubayashi T. (2011). Strenuous resistance exercise effects on magnetic resonance diffusion parameters and muscle-tendon function in human skeletal muscle. J. Magn. Reson. Imaging 34, 887–894. 10.1002/jmri.22668, PMID: 21769968

[ref41] Zaraiskaya T. Kumbhare D. Noseworthy M. D. (2006). Diffusion tensor imaging in evaluation of human skeletal muscle injury. J. Magn. Reson. Imaging 24, 402–408. 10.1002/jmri.20651, PMID: 16823776

[ref42] Zhang L. Y. Ding J. T. Wang Y. Zhang W. G. Deng X. J. Chen J. H. (2011). MRI quantitative study and pathologic analysis of crush injury in rabbit hind limb muscles. J. Surg. Res. 167, e357–e363. 10.1016/j.jss.2010.09.014, PMID: 21035134

[ref43] Zhao D. M. Zhu S. Q. Wang F. R. Huang S. S. (2019). Role of mutant TBP in regulation of myogenesis on muscle satellite cells. Curr. Med. Sci. 39, 734–740. 10.1007/s11596-019-2099-y, PMID: 31612390

