# Supplementary figures and images for: A Longitudinal Study of T2 Mapping Combined With Diffusion Tensor Imaging to Quantitatively Evaluate Tissue Repair of Rat Skeletal Muscle After Frostbite
Source: Front Physiol. 2021 Jan 25;11:597638. doi: 10.3389/fphys.2020.597638 (PMC7868413; doi:10.3389/fphys.2020.597638)

GAPDH

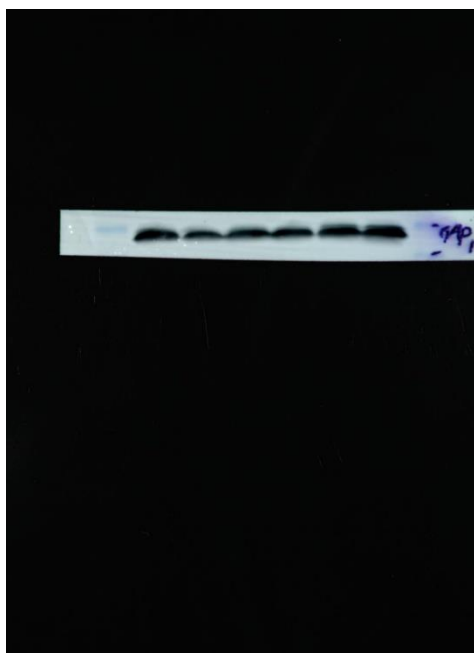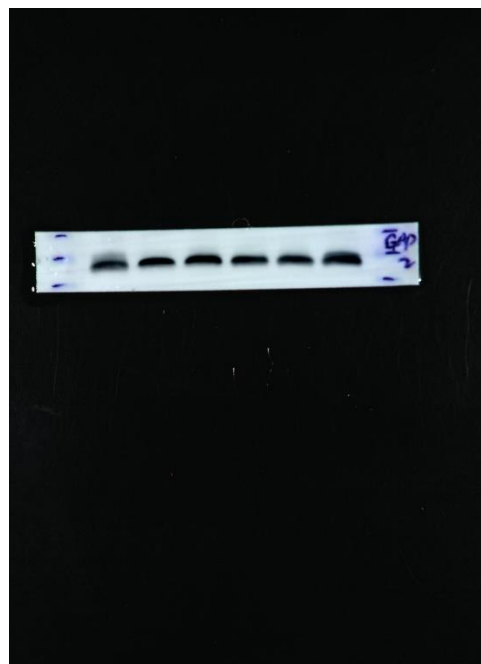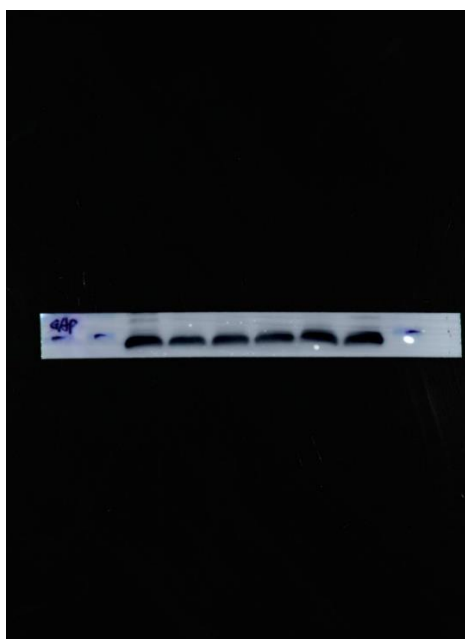

Myod1

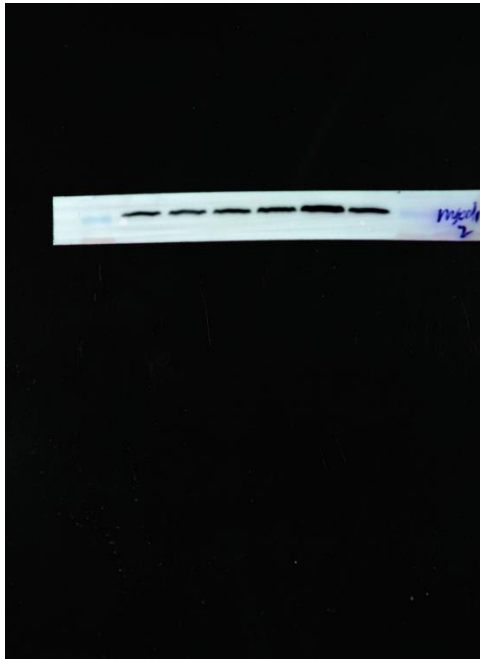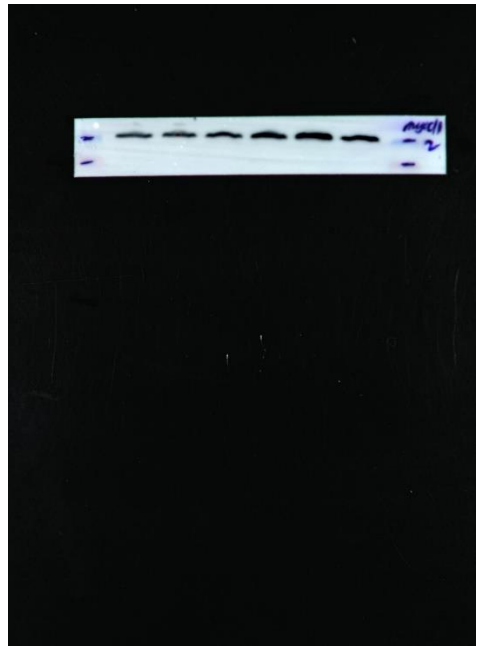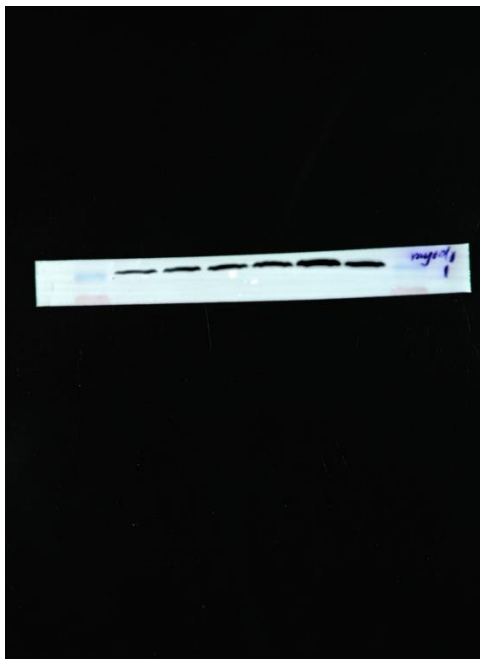

TNF- $\alpha$

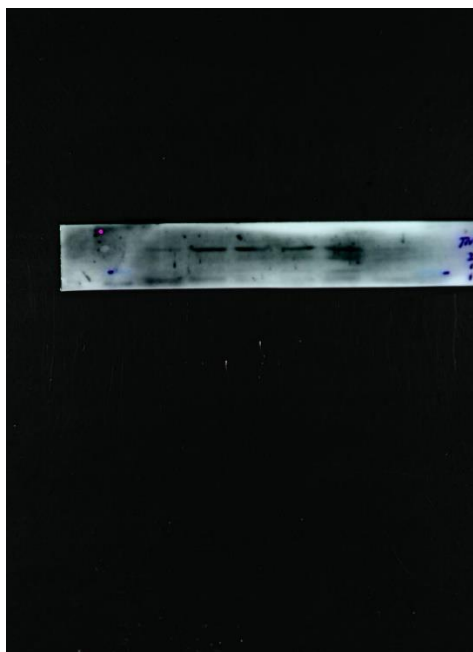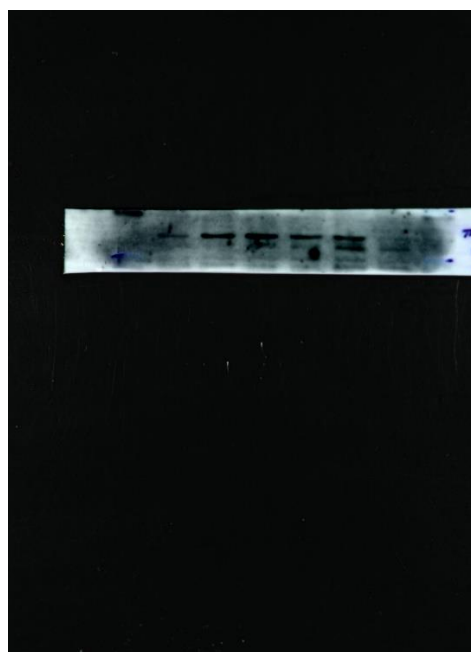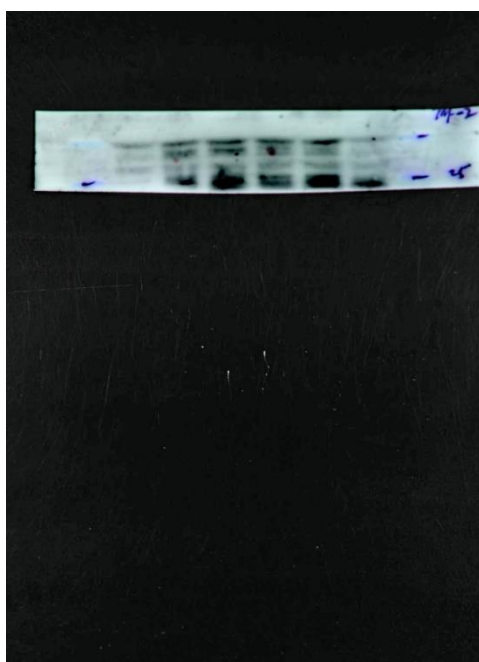

Supplement: Supplementary file 1 [file Data_Sheet_1.pdf]
